# Existence and exponential stability of positive almost periodic solution for Nicholson’s blowflies models on time scales

**DOI:** 10.1186/s40064-016-2700-9

**Published:** 2016-07-16

**Authors:** Yongkun Li, Bing Li

**Affiliations:** Department of Mathematics, Yunnan University, Kunming, 650091 Yunnan People’s Republic of China

**Keywords:** Almost periodic solution, Exponential stability, Nicholsons blowflies model, Almost periodic time scales, 34N05, 34K14, 34K20, 92D25

## Abstract

In this paper, we first give a new definition of almost periodic time scales, two new definitions of almost periodic functions on time scales and investigate some basic properties of them. Then, as an application, by using a fixed point theorem in Banach space and the time scale calculus theory, we obtain some sufficient conditions for the existence and exponential stability of positive almost periodic solutions for a class of Nicholson’s blowflies models on time scales. Finally, we present an illustrative example to show the effectiveness of obtained results. Our results show that under a simple condition the continuous-time Nicholson’s blowflies model and its discrete-time analogue have the same dynamical behaviors.

## Background

To describe the population of the Australian sheep-blowfly and to agree with the experimental data obtained in Nicholson (1954), Gurney et al. (1980) proposed the following delay differential Equation model:

1 $$\begin{aligned} x'(t)=-\delta x(t)+px(t-\tau )e^{-ax(t-\tau )}, \end{aligned}$$

where *p* is the maximum per capita daily egg production rate, 1 / *a* is the size at which the blowfly population reproduces at its maximum rate, $\delta$ is the per capita daily adult death rate, and $\tau$ is the generation time. Since Eq. (1) explains Nicholson’s data of blowfly more accurately, the model and its modifications have been now refereed to as Nicholson’s Blowflies model. The theory of the Nicholson’s blowflies equation has made a remarkable progress in the past 40 years with main results scattered in many research papers. Many important results on the qualitative properties of the model such as the existence of positive solutions, positive periodic solutions, positive almost periodic solutions and positive pseudo almost periodic solutions, the persistence, the permanence, the oscillation and the stability for the classical Nicholson’s model and its generalizations have been established in the literature (Chen 2003; Li and Du 2008; Liu 2010, 2014a; Saker and Agarwal 2002; Zhou 2013; Yi and Zou 2008; Liu and Gong 2011; Hien 2014; Chérif 2015; Duan and Huang 2015; Yao 2015a; Shao 2012). For example, to describe the models of marine protected areas and B-cell chronic lymphocytic leukemia dynamics that are examples of Nicholson-type delay differential systems, Berezansky et al. (2011) and Wang et al. (2011) studied the following Nicholson-type delay system:

$$\left\{ \begin{array}{lll} N'_1(t) =-\alpha _1(t)N_1(t)+\beta _1(t)N_2(t)+\sum \limits ^{m}_{j=1}c_{1j}(t)N_1(t-\tau _{1j}(t))e^{-\gamma _{ij}(t)N_1(t-\tau _{1j}(t))} ,\\ N'_2(t) =-\alpha _2(t)N_2(t)+\beta _2(t)N_1(t)+\sum \limits ^{m}_{j=1}c_{2j}(t)N_2(t-\tau _{1j}(t))e^{-\gamma _{ij}(t)N_2(t-\tau _{1j}(t))} , \end{array} \right.$$

where $\alpha _i, \beta _i, c_{ij}, \gamma _{ij}, \tau _{ij}\in C(\mathbb {R}, (0,+\infty ))$, $i=1,2, j=1,2,\ldots ,m$; in Faria (2011), the authors discussed some aspects of the global dynamics for a Nicholson’s blowflies model with patch structure given by

$$\begin{aligned} x'_i(t)=-d_ix_i(t)+\sum \limits _{j=1}^na_{ij}x_j(t) +\sum \limits _{j=1}^m\beta _{ij}x_i(t-\tau _{ij})e^{-x_i(t-\tau _{ij})}, \quad i=1,2,\ldots ,n. \end{aligned}$$

In the real world phenomena, since the almost periodic variation of the environment plays a crucial role in many biological and ecological dynamical systems and is more frequent and general than the periodic variation of the environment. Hence, the effects of almost periodic environment on evolutionary theory have been the object of intensive analysis by numerous authors and some of these results for the Nicholson’s blowflies model can be found in Alzabut (2010), Chen and Liu (2011), Long (2012), Wang (2013), Liu and Meng (2012), Xu (2014), Liu (2014b), Ding and Alzabut (2015).

Besides, although most models are described by differential equations, the discrete-time models governed by difference equations are more appropriate than the continuous ones when the size of the population is rarely small, or the population has non-overlapping generations. Hence, it is also important to study the dynamics of the discrete-time Nicholson’s blowflies model. Recently, authors of Yao (2014), Alzabut (2013) studied the existence and exponential convergence of almost periodic solutions for the discrete Nicholson’s blowflies model, respectively. In fact, it is troublesome to study the dynamics for continuous systems and their corresponding discrete ones respectively, therefore, it is significant to study that on time scales, which was initiated by Stefan Hilger (see Hilger 1990) in order to unify continuous and discrete cases. However, to the best of our knowledge, very few results are available on the existence and stability of positive almost periodic solutions for the Nicholson’s blowflies model on time scales except (Li and Yang 2012). But Li and Yang (2012) only considered the asymptotical stability of the model and the exponential stability is stronger than asymptotical stability among different stabilities.

On the other hand, in order to study the almost periodic dynamic equations on time scales, a concept of almost periodic time scales was proposed in Li and Wang (2011a). Based on this concept, almost periodic functions Li and Wang (2011a), pseudo almost periodic functions (Li and Wang 2012), almost automorphic functions (Lizama and Mesquita 2013a), weighted pseudo almost automorphic functions (Wang and Li 2013), weighted piecewise pseudo almost automorphic functions (Wang and Agarwal 2014a) and almost periodic set-valued functions (Hong and Peng 2016) on on time scales were defined successively. Also, some works have been done under the concept of almost periodic time scales (see Lizama and Mesquita 2013b; Lizama et al. 2014; Li and Yang 2014; Liang et al. 2014; Gao et al. 2014; Yao 2015b; Mophou et al. 2014; Zhou et al. 2015). Although the concept of almost periodic time scales in Li and Wang (2011a) can unify the continuous and discrete situations effectively, it is very restrictive. This excludes many interesting time scales. Therefore, it is a challenging and important problem in theories and applications to find new concepts of almost periodic time scales (Li and Wang 2011b; Wang and Agarwal 2014b; Li and Li 2015; Li et al. 2015a, b).

Motivated by the above discussion, our main purpose of this paper is firstly to propose a new definition of almost periodic time scales, two new definitions of almost periodic functions on time scales and study some basic properties of them. Then, as an application, we study the existence and global exponential stability of positive almost periodic solutions for the following Nicholson’s blowflies model with patch structure and multiple time-varying delays on time scales:

2 $$\begin{aligned} x_i^\Delta (t)& = -c_i(t)x_i(t)+\sum \limits _{k=1,k\ne i}^nb_{ik}(t)x_k(t)\nonumber \\&\quad +\sum \limits _{j=1}^n\beta _{ij}(t)x_i(t-\tau _{ij}(t))e^{-\alpha _{ij}(t)x_i(t-\tau _{ij}(t))},\quad i=1,2,\ldots ,n, \end{aligned}$$

where $t\in \mathbb {T}$, $\mathbb {T}$ is an almost periodic time scale, $x_i(t)$ denotes the density of the species in patch *i*, $b_{ik}(k\ne i)$ is the migration coefficient from patch *k* to patch *i* and the natural growth in each patch is of Nicholson-type.

For convenience, for a positive almost periodic function $f:\mathbb {T}\rightarrow \mathbb {R}$, we denote $f^+=\sup \nolimits _{t\in \mathbb {T}}f(t), f^-=\inf \nolimits _{t\in \mathbb {T}}f(t)$. Due to the biological meaning of (2), we just consider the following initial condition:

3 $$\begin{aligned} \varphi _i(s)>0,\,\,s\in [t_0-\theta ,t_0]_{\mathbb {T}},\,t_0\in \mathbb {T},\quad i=1,2,\ldots ,n, \end{aligned}$$

where $$\theta =\max \nolimits _{(i,j)}\sup \nolimits _{t\in \mathbb {T}}\{\tau _{ij}(t)\}$$, $$[t_0-\theta ,t_0]_{\mathbb {T}}=[t_0-\theta ,t_0]\cap \mathbb {T}$$.

This paper is organized as follows: In “Preliminaries”, we introduce some notations and definitions which are needed in later sections. In “Almost periodic time scales and almost periodic functions on time scales” section, we give a new definition of almost periodic time scales and two new definitions of almost periodic functions on time scales, and we state and prove some basic properties of them. In “Positive almost periodic solutions for the Nicholson’s blowflies model” section, we establish some sufficient conditions for the existence and exponential stability of positive almost periodic solutions of (2). In “An example” section, we give an example to illustrate the feasibility of our results obtained in previous sections. We draw a conclusion in “Conclusion” section.

## Preliminaries

In this section, we shall first recall some definitions and state some results which are used in what follows.

Let $\mathbb {T}$ be a nonempty closed subset (time scale) of $\mathbb {R}$. The forward and backward jump operators $\sigma , \rho :\mathbb {T}\rightarrow \mathbb {T}$ and the graininess $\mu :\mathbb {T}\rightarrow \mathbb {R}^+$ are defined, respectively, by

$$\begin{aligned} \sigma (t)=\inf \{s\in \mathbb {T}:s>t\},\quad \rho (t)=\sup \{s\in \mathbb {T}:s<t\}\quad \hbox {and} \quad \mu (t)=\sigma (t)-t. \end{aligned}$$

A point $t\in \mathbb {T}$ is called left-dense if $t>\inf \mathbb {T}$ and $\rho (t)=t$, left-scattered if $\rho (t)<t$, right-dense if $t<\sup \mathbb {T}$ and $\sigma (t)=t$, and right-scattered if $\sigma (t)>t$. If $\mathbb {T}$ has a left-scattered maximum *m*, then $\mathbb {T}^k=\mathbb {T}{\setminus} \{m\}$; otherwise $\mathbb {T}^k=\mathbb {T}$. If $\mathbb {T}$ has a right-scattered minimum *m*, then $\mathbb {T}_k=\mathbb {T}{\setminus} \{m\}$; otherwise $\mathbb {T}_k=\mathbb {T}$.

A function $f:\mathbb {T}\rightarrow \mathbb {R}$ is right-dense continuous provided it is continuous at right-dense point in $\mathbb {T}$ and its left-side limits exist at left-dense points in $\mathbb {T}$. If *f* is continuous at each right-dense point and each left-dense point, then *f* is said to be continuous function on $\mathbb {T}$.

For $y:\mathbb {T}\rightarrow \mathbb {R}$ and $t\in \mathbb {T}^k$, we define the delta derivative of *y*(*t*), $y^\Delta (t)$, to be the number (if it exists) with the property that for a given $\varepsilon >0$, there exists a neighborhood *U* of *t* such that

$$\begin{aligned} |[y(\sigma (t))-y(s)]-y^\Delta (t)[\sigma (t)-s]|<\varepsilon |\sigma (t)-s| \end{aligned}$$

for all $$s\in U$$.

If *y* is continuous, then *y* is right-dense continuous, and if *y* is delta differentiable at *t*, then *y* is continuous at *t*.

Let *y* be right-dense continuous. If $Y^{\Delta }(t)=y(t)$, then we define the delta integral by $\int _a^{t}y(s)\Delta s=Y(t)-Y(a).$

A function $r:\mathbb {T}\rightarrow \mathbb {R}$ is called regressive if $1+\mu (t)r(t)\ne 0$ for all $t\in \mathbb {T}^k$. The set of all regressive and *rd*-continuous functions $r:\mathbb {T}\rightarrow \mathbb {R}$ will be denoted by $\mathcal {R}=\mathcal {R}(\mathbb {T})=\mathcal {R}(\mathbb {T},\mathbb {R})$. We define the set $\mathcal {R}^+=\mathcal {R}^+(\mathbb {T},\mathbb {R})=\{r\in \mathcal {R}:1+\mu (t)r(t)>0,\,\,\forall t\in \mathbb {T}\}$.

### **Lemma 1**

(Bohner and Peterson 2001) *Suppose that*$p\in \mathcal {R}^{+}$, *then*

*(i)*: $$e_{p}(t,s)>0$$, *for all*$$t,s\in \mathbb {T}$$;
(*ii*): *if*$p(t)\le q(t)$*for all*$t\ge s, t,s\in \mathbb {T}$, *then*$e_{p}(t,s)\le e_{q}(t,s)$*for all*$t \ge s$.

### **Definition 1**

(Fink 1974) A subset *S* of $\mathbb {R}$ is called relatively dense if there exists a positive number *L* such that $[a, a + L] \cap S \ne \phi$ for all $a\in \mathbb {R}$. The number *L* is called the inclusion length.

### **Definition 2**

(Li and Wang 2011a) A time scale $\mathbb {T}$ is called an almost periodic time scale if

$$\begin{aligned} \Pi =\big \{\tau \in \mathbb {R}: t\pm \tau \in \mathbb {T}, \forall t\in {\mathbb {T}}\big \}\ne \{0\}. \end{aligned}$$

The following definition is a slightly modified version of Definition 3.10 in Li and Wang (2011a).

### **Definition 3**

Let $\mathbb {T}$ be an almost periodic time scale. A function $f\in C(\mathbb {T}\times D,\mathbb {E}^n)$ is called an almost periodic function in $t\in \mathbb {T}$ uniformly for $x\in D$ if the $\varepsilon$-translation set of *f*

$$\begin{aligned} E\{\varepsilon ,f,S\}=\{\tau \in \Pi :|f(t+\tau ,x)-f(t,x)|<\varepsilon ,\quad \forall (t,x)\in \mathbb {T}\times S\} \end{aligned}$$

is relatively dense for all $\varepsilon >0$ and for each compact subset *S* of *D*; that is, for any given $\varepsilon >0$ and each compact subset *S* of *D*, there exists a constant $l(\varepsilon ,S)>0$ such that each interval of length $l(\varepsilon ,S)$ contains a $\tau (\varepsilon ,S)\in E\{\varepsilon ,f,S\}$ such that

$$\begin{aligned} |f(t+\tau ,x)-f(t,x)|<\varepsilon , \quad \forall (t,x)\in \mathbb {T}\times S. \end{aligned}$$

$\tau$ is called the $\varepsilon$-translation number of *f*.

## Almost periodic time scales and almost periodic functions on time scales

In this section, we will give a new definition of almost periodic time scales and two new definitions of almost periodic functions on time scales, and we will investigate some basic properties of them. Our new definition of almost periodic time scales is as follows:

### **Definition 4**

A time scale $\mathbb {T}$ is called an almost periodic time scale if the set

$$\begin{aligned} \Pi_0 :=\left \{\tau \in \mathbb {R}:\mathbb {T}_{\pm\tau} \ne \emptyset \right \}\ne \{0\}, \end{aligned}$$

where $\mathbb {T}_\tau =\mathbb {T}\cap \{\mathbb {T}-\tau \}=\mathbb {T}\cap \{t-\tau : t\in \mathbb {T}\}$, and there exists a set Π_1 _satisfies

(i): $$0\in\Pi_1\subseteq\Pi_0$$,
(ii): $$\Pi(\Pi_1)\setminus\{0\}\neq \emptyset$$ ,
(iii): $$\widetilde{\mathbb {T}}:=\mathbb {T}(\Pi )=\bigcap \nolimits _{\tau \in \Pi }\mathbb {T}_\tau \ne \emptyset$$,

where $$\Pi:=\Pi(\Pi_1)=\{\tau\in \Pi_1\subseteq \Pi_0: \sigma\pm\tau\in \Pi_1, \forall \sigma\in \Pi_1\big\}$$.

Clearly, if $t\in \mathbb {T}_\tau$, then $t+\tau \in \mathbb {T}.$ If $t\in \widetilde{\mathbb {T}}$, then $t+\tau \in \mathbb {T}$ for $\tau \in \Pi$.

### *Remark 1*

Obviously, if $\mathbb {T}$ is an almost periodic time scale under Definition 4, then $\inf \mathbb {T}=-\infty$ and $\sup \mathbb {T}=+\infty .$ If $\mathbb {T}$ is an almost periodic time scale under Definition 2, then $\mathbb {T}$ is also an almost periodic time scale under Definition 4 and in this case, $\widetilde{\mathbb {T}}=\mathbb {T}$.

### *Example 1*

Let $\mathbb {T}=\mathbb {Z}\cup \{\frac{1}{4}\}$. $\mathrm{Take}\,\, \Pi_1=\big\{\tau\in \mathbb{T}:\mathbb{T}_\tau\neq \emptyset, \, \mathbb{T}_\tau\neq \{0\}\big\}\subseteq\Pi_0, \,\,\mathrm{then}\,\,\mathrm{for}\,\, \mathrm{every} \,\,\tau\in \mathbb{Z}$, we have $\mathbb {T}_{\tau }=\mathbb {Z}$ and $\mathbb {T}_{\frac{1}{4}}=\{0\}$. Hence $\Pi =\mathbb {Z}$ and $\widetilde{\mathbb {T}}=\bigcap _{\tau \in \Pi }\mathbb {T}_{\tau }=\mathbb {Z}\ne \emptyset$. So, $\mathbb {T}$ is an almost periodic time scale under Definition 4 but it is not an almost periodic time scale under Definition 2.

### **Lemma 2**

*If*$\mathbb {T}$*is an almost periodic time scales under Definition 4*, *then*$\widetilde{\mathbb {T}}$*is an almost periodic time scale under Definition 2*.

### *Proof*

By contradiction, suppose that there exists a $t_0\in \widetilde{\mathbb {T}}$ such that for every $\tau \in \Pi {\setminus} \{0\}$, $t_0+\tau \notin \widetilde{\mathbb {T}}$ or $t_0-\tau \notin \widetilde{\mathbb {T}}$.

**Case (i)** If $t_0+\tau \notin \widetilde{\mathbb {T}}$, then there exists a ${\tau _{t_0}}\in \Pi$ such that $t_0+\tau \notin \mathbb {T}_{{\tau _{t_0}}}$. On one hand, since $t_0+\tau \in \mathbb {T}$, $t_0+\tau +\tau _{t_0}\notin \mathbb {T}$. On the other hand, since $t_0\in \widetilde{\mathbb {T}}$ and $\tau +\tau _{t_0}\in \Pi$, $t_0+\tau +\tau _{t_0}\in \mathbb {T}$. This is a contradiction.

**Case (ii)** If $t_0-\tau \notin \widetilde{\mathbb {T}}$, then there exists a ${\tilde{\tau }_{t_0}}\in \Pi$ such that $t_0-\tau \notin \mathbb {T}_{{\tilde{\tau }_{t_0}}}$. On one hand, since $t_0-\tau \in \mathbb {T}$, $t_0-\tau +\tilde{\tau }_{t_0}\notin \mathbb {T}$. On the other hand, since $t_0\in \widetilde{\mathbb {T}}$ and $-\tau +\tilde{\tau }_{t_0}\in \Pi$, $t_0-\tau +\tilde{\tau }_{t_0}\in \mathbb {T}$. This is a contradiction.

Therefore, for every $t\in \widetilde{\mathbb {T}}$, there exists a $\tau \in \Pi {\setminus} \{0\}$ such that $t\pm \tau \in \widetilde{\mathbb {T}}$. Hence, $\mathbb {T}$ is an almost periodic time scale under Definition 2. The proof is complete. $\square$

Throughout this section, $\mathbb {E}^{n}$ denotes $\mathbb {R}^{n}$ or $\mathbb {C}^{n}$, *D* denotes an open set in $\mathbb {E}^{n}$ or $D=\mathbb {E}^{n}$, *S* denotes an arbitrary compact subset of *D*.

From Li and Wang (2011a), under Definitions 2 and 3, we know that if we denote by $BUC(\mathbb {T}\times D,\mathbb {R}^{n})$ the collection of all bounded uniformly continuous functions from $\mathbb {T}\times S$ to $\mathbb {R}^{n}$, then

4 $$\begin{aligned} AP(\mathbb {T}\times D,\mathbb {R}^{n})\subset BUC(\mathbb {T}\times D,\mathbb {R}^{n}), \end{aligned}$$

where $AP(\mathbb {T}\times D,\mathbb {R}^{n})$ are the collection of all almost periodic functions in $t\in \mathbb {T}$ uniformly for $x\in D$. It is well known that if we let $\mathbb {T}=\mathbb {R}$ or $\mathbb {Z}$, (4) is valid. So, for simplicity, we give the following definition:

### **Definition 5**

Let $\mathbb {T}$ be an almost periodic time scale under sense of Definition 4. A function $f\in BUC(\mathbb {T}\times D,\mathbb {E}^n)$ is called an almost periodic function in $t\in \mathbb {T}$ uniformly for $x\in D$ if the $\varepsilon$-translation set of *f*

$$\begin{aligned} E\{\varepsilon ,f,S\}=\{\tau \in \Pi :|f(t+\tau ,x)-f(t,x)|<\varepsilon ,\quad \forall (t,x)\in \widetilde{\mathbb {T}}\times S\} \end{aligned}$$

is relatively dense for all $\varepsilon >0$ and for each compact subset *S* of *D*; that is, for any given $\varepsilon >0$ and each compact subset *S* of *D*, there exists a constant $l(\varepsilon ,S)>0$ such that each interval of length $l(\varepsilon ,S)$ contains a $\tau (\varepsilon ,S)\in E\{\varepsilon ,f,S\}$ such that

$$\begin{aligned} |f(t+\tau ,x)-f(t,x)|<\varepsilon , \quad \forall (t,x)\in \widetilde{\mathbb {T}}\times S. \end{aligned}$$

This $\tau$ is called the $\varepsilon$-translation number of *f*.

### *Remark 2*

If $\mathbb {T}=\mathbb {R}$, then $\widetilde{\mathbb {T}}=\mathbb {R}$, in this case, if we take $\Pi=\mathbb {R}$, then Definition 5 is actually equivalent to the definition of the uniformly almost periodic functions in Ref. Fink (1974). If $\mathbb {T}=\mathbb {Z}$, then $\widetilde{\mathbb {T}}=\mathbb {Z}$, in this case, if we take $\Pi=\mathbb {Z}$, then Definition 5 is actually equivalent to the definition of the uniformly almost periodic sequences in Fink and Seifert (1969), David and Cristina (2004).

### *Example 2*

Let $\mathbb {T}=\mathbb {Z}\cup \{\frac{1}{4}\}$, according to Example 3.1, $\mathbb {T}$ is an almost periodic time scale under Definition 4. Take $f(t,x)=2x^2+\sin 2 t+\cos \sqrt{3} t$ for $(t,x)\in \mathbb {T}\times \mathbb {R}$. Then *f* is an almost periodic function in $t\in \mathbb {T}$ uniformly for $x\in \mathbb {R}$ under Definition 5.

For convenience, we denote by $AP(\mathbb {T}\times D,\mathbb {E}^n)$ the set of all functions that are almost periodic in *t* uniformly for $x\in D$ and denote by $AP(\mathbb {T})$ the set of all functions that are almost periodic in $t\in \mathbb {T}$, and introduce some notations: Let $\alpha =\{\alpha _{n}\}$ and $\beta =\{\beta _{n}\}$ be two sequences. Then $\beta \subset \alpha$ means that $\beta$ is a subsequence of $\alpha$; $\alpha +\beta =\{\alpha _{n}+\beta _{n}\}; -\alpha =\{-\alpha _{n}\}$; and $\alpha$ and $\beta$ are common subsequences of $\alpha ^{'}$ and $\beta ^{'}$, respectively, means that $\alpha _{n}=\alpha ^{'}_{n(k)}$ and $\beta _{n}=\beta ^{'}_{n(k)}$ for some given function *n*(*k*). We introduce the translation operator *T*, $T_{\alpha }f(t,x)=g(t,x)$ means that $g(t,x)= \lim \limits _{n\rightarrow +\infty }f(t+\alpha _{n},x)$ and is written only when the limit exists. The mode of convergence, e.g. pointwise, uniform, etc., will be specified at each use of the symbol.

Similar to the proofs of Theorem 3.14, Theorem 3.21 and Theorem 3.22 in Li and Wang (2011a), respectively, one can prove the following three theorems.

### **Theorem 1**

*Let*$f\in UBC(\mathbb {T}\times D,\mathbb {E}^{n}),$*if for any sequence*$\alpha ^{'}\subset \Pi$, *there exists*$\alpha \subset \alpha ^{'}$*such that*$T_{\alpha }f$*exists uniformly on*$\widetilde{\mathbb {T}}\times S$, *then*$f\in AP(\mathbb {T}\times D,\mathbb {E}^n)$.

### **Theorem 2**

*If*$f\in AP(\mathbb {T}\times D,\mathbb {E}^n)$, *then for any*$\varepsilon >0$, *there exists a positive constant*$L=L(\varepsilon ,S)$, *for any*$a\in \mathbb {R}$, *there exist a constant*$\eta >0$*and*$\alpha \in \mathbb {R}$*such that*$\big ([\alpha ,\alpha +\eta ]\cap \Pi \big )\subset [a,a+L]$*and*$\big ([\alpha ,\alpha +\eta ]\cap \Pi \big )\subset E(\varepsilon ,f,S)$.

### **Theorem 3**

*If*$f,g\in AP(\mathbb {T}\times D,\mathbb {E}^n)$, *then for any*$\varepsilon >0$, $E(f,\varepsilon ,S)\cap E(g,\varepsilon ,S)$*is nonempty relatively dense*.

According to Definition 5, one can easily prove

### **Theorem 4**

*If*$f\in AP(\mathbb {T}\times D,\mathbb {E}^n)$, *then for any*$\alpha \in \mathbb {R}, b\in \Pi$, *functions*$\alpha f,f(t+b,\cdot )\in AP(\mathbb {T}\times D,\mathbb {E}^n)$.

Similar to the proofs of Theorem 3.24, Theorem 3.27, Theorem 3.28 and Theorem 3.29 in Li and Wang (2011a), respectively, one can prove the following four theorems.

### **Theorem 5**

*If*$f,g\in AP(\mathbb {T}\times D,\mathbb {E}^n)$, *then*$f+g,fg\in AP(\mathbb {T}\times D,\mathbb {E}^n)$, *if*   $\inf \limits _{t\in \mathbb {T}}|g(t,x)|>0$, *then*${f}/{g}\in AP(\mathbb {T}\times D,\mathbb {E}^n)$.

### **Theorem 6**

*If*$f_{n}\in AP(\mathbb {T}\times D,\mathbb {E}^{n}) (n=1,2,\ldots )$*and the sequence*$\{f_{n}\}$*uniformly converges tofon*$\mathbb {T}\times S$, *then*$f\in AP(\mathbb {T}\times D,\mathbb {E}^n)$.

### **Theorem 7**

*If*$f\in AP(\mathbb {T}\times D,\mathbb {E}^n)$, *denote*$F(t,x)=\int _{0}^{t}f(s,x)\Delta s,$*then*$F\in AP(\mathbb {T}\times D,\mathbb {E}^n)$*if and only ifFis bounded on*$\mathbb {T}\times S$.

### **Theorem 8**

*If*$f\in AP(\mathbb {T}\times D,\mathbb {E}^n)$, $F(\cdot )$*is uniformly continuous on the value field off*, *then*$F\circ f$*is almost periodic intuniformly for*$x\in D$.

By Definition 5, one can easily prove

### **Theorem 9**

*Let*$f:\mathbb {R}\rightarrow \mathbb {R}$*satisfies Lipschitz condition and*$\varphi (t)\in AP(\mathbb {T})$, *then*$f(\varphi (t))\in AP(\mathbb {T})$.

### **Definition 6**

(Li and Wang 2011b) Let *A*(*t*) be an $n\times n$ rd-continuous matrix on $\mathbb {T}$, the linear system

5 $$\begin{aligned} x^{\Delta }(t)=A(t)x(t), \quad t\in \mathbb {T} \end{aligned}$$

is said to admit an exponential dichotomy on $\mathbb {T}$ if there exist positive constant *k*, $\alpha$, projection *P*, and the fundamental solution matrix *X*(*t*) of (5), satisfying

$$\begin{aligned}&|X(t)PX^{-1}(\sigma (s))|\le k e_{\ominus _\alpha }(t, \sigma (s)),\,\,s,t\in \mathbb {T},\,\, t\ge \sigma (s),\\&\quad |X(t)(I-P)X^{-1}(\sigma (s))|\le k e_{\ominus _\alpha }(\sigma (s),t),\,\,s,t\in \mathbb {T},\,\, t\le \sigma (s), \end{aligned}$$

where $|\cdot |$ is a matrix norm on $\mathbb {T}$, that is, if $A=(a_{ij})_{n\times n}$, then we can take $|A|=\big (\sum \nolimits _{i=1}^n\sum \nolimits _{j=1}^n|a_{ij}|^2\big )^{\frac{1}{2}}$.

Similar to the proof of Lemma 2.15 in Li and Wang (2011b), one can easily show that

### **Lemma 3**

*Let*$a_{ii}(t)$*be an uniformly boundedrd-continuous function on*$\mathbb {T}$, *where*$a_{ii}(t)>0$, $-a_{ii}(t)\in \mathcal {R}^{+}$*for every*$t\in \mathbb {T}$*and*

$$\begin{aligned} \min _{1\le i\le n}\{\inf _{t\in \mathbb {T}}a_{ii}(t)\}>0, \end{aligned}$$

*then the linear system*

$$\begin{aligned} x^{\Delta }(t)=diag(-a_{11}(t), -a_{22}(t), \ldots , -a_{nn}(t))x(t) \end{aligned}$$

*admits an exponential dichotomy on*$\mathbb {T}$.

According to Lemma 2, $\widetilde{\mathbb {T}}$ is an almost periodic time scales under Definition 2, we denote the forward and the backward jump operators of $\widetilde{\mathbb {T}}$ by $\widetilde{\sigma }$ and $\widetilde{\rho }$, respectively.

### **Lemma 4**

*Iftis a right-dense point of*$\widetilde{\mathbb {T}}$, *thentis also a right-dense point of*$\mathbb {T}$.

### *Proof*

Let *t* be a right-dense point of $\widetilde{\mathbb {T}}$, then

$$\begin{aligned} t=\widetilde{\sigma }(t)=\inf \{s\in \widetilde{\mathbb {T}}:s>t\}\ge \inf \{s\in \mathbb {T}:s>t\}=\sigma (t). \end{aligned}$$

Since $\sigma (t)\ge t$, $t=\sigma (t)$. The proof is complete. $\square$

Similar to the proof of Lemma 4, one can prove the following lemma.

### **Lemma 5**

*Iftis a left-dense point of*$\widetilde{\mathbb {T}}$, *thentis also a left-dense point of*$\mathbb {T}$.

For each $f\in C(\mathbb {T},\mathbb {R})$, we define $\widetilde{f}:\widetilde{\mathbb {T}}\rightarrow \mathbb {R}$ by $\widetilde{f}(t)=f(t)$ for $t\in \widetilde{\mathbb {T}}$. From Lemmas 4 and  5, we can get that $\widetilde{f}\in C(\widetilde{\mathbb {T}},\mathbb {R})$. Therefore, *F* defined by

$$\begin{aligned} F(t):=\int ^{t}_{t_0}\widetilde{f}(\tau )\widetilde{\Delta }\tau ,\,\, t_0,t\in \widetilde{\mathbb {T}} \end{aligned}$$

is an antiderivative of *f* on $\widetilde{\mathbb {T}}$, where $\widetilde{\Delta }$ denotes the $\Delta$-derivative on $\widetilde{\mathbb {T}}$.

Set $\widetilde{\Pi }=\{\tau \in \Pi : t\pm \tau \in \widetilde{\mathbb {T}}\}$. We give our second definition of almost periodic functions on time scales as follows.

### **Definition 7**

Let $\mathbb {T}$ be an almost periodic time scale under sense of Definition 4. A function $f\in BUC(\mathbb {T}\times D,\mathbb {E}^n)$ is called an almost periodic function in $t\in \mathbb {T}$ uniformly for $x\in D$ if the $\varepsilon$-translation set of *f*

$$\begin{aligned} E\{\varepsilon ,f,S\}=\{\tau \in \widetilde{\Pi }:|f(t+\tau ,x)-f(t,x)|<\varepsilon ,\quad \forall (t,x)\in \widetilde{\mathbb {T}}\times S\} \end{aligned}$$

is relatively dense for all $\varepsilon >0$ and for each compact subset *S* of *D*; that is, for any given $\varepsilon >0$ and each compact subset *S* of *D*, there exists a constant $l(\varepsilon ,S)>0$ such that each interval of length $l(\varepsilon ,S)$ contains a $\tau (\varepsilon ,S)\in E\{\varepsilon ,f,S\}$ such that

$$\begin{aligned} |f(t+\tau ,x)-f(t,x)|<\varepsilon , \quad \forall (t,x)\in \widetilde{\mathbb {T}}\times S. \end{aligned}$$

This $\tau$ is called the $\varepsilon$-translation number of *f*.

### *Remark 3*

It is clear that if a function is an almost periodic function under Definition 5, then it is also an almost periodic function under Definition 7.

### *Remark 4*

Since $\widetilde{\mathbb {T}}$ is an almost periodic time scales under Definition 2, under Definition 5, all the results obtained in Li and Wang (2011a) remain valid when we restrict our discussion to $\widetilde{\mathbb {T}}$.

In the following, we restrict our discuss under Definition 7.

Consider the following almost periodic system:

6 $$\begin{aligned} x^{\Delta }(t)=A(t)x(t)+f(t), \quad t\in \mathbb {T}, \end{aligned}$$

where *A*(*t*) is a $n\times n$ almost periodic matrix function, *f*(*t*) is a *n*-dimensional almost periodic vector function.

Similar to Lemma 2.13 in Li and Wang (2011b), one can easily get

### **Lemma 6**

*If linear system* (5) *admits an exponential dichotomy, then system* (6) *has a bounded solutionx*(*t*) *as follows:*

$$\begin{aligned} x(t)=\int _{-\infty }^{t}X(t)PX^{-1}(\sigma (s))f(s)\Delta s-\int ^{+\infty }_{t}X(t)(I-P)X^{-1}(\sigma (s))f(s)\Delta s,\,\, t\in \mathbb {T}, \end{aligned}$$

*whereX*(*t*) *is the fundamental solution matrix of* (5).

By Theorem 4.19 in Li and Wang (2011a), we have

### **Lemma 7**

*LetA*(*t*) *be an almost periodic matrix function andf*(*t*) *be an almost periodic vector function. If* (5) *admits an exponential dichotomy, then* (6) *has a unique almost periodic solution:*

$$\begin{aligned} x(t)=\int ^t_{-\infty }\widetilde{X}(t)P\widetilde{X}^{-1}(\widetilde{\sigma }(s))\widetilde{f}(s)\widetilde{\Delta } s-\int _t^{+\infty }\widetilde{X}(t)(I-P)\widetilde{X}^{-1}(\widetilde{\sigma }(s))\widetilde{f}(s)\widetilde{\Delta } s,\,\, t\in \widetilde{\mathbb {T}}, \end{aligned}$$

*where*$\widetilde{X}(t)$*is the restriction of the fundamental solution matrix of* (5) *on*$\widetilde{\mathbb {T}}$.

From Definition 5 and Lemmas 6 and 7, one can easily get the following lemma.

### **Lemma 8**

*If linear system* (5) *admits an exponential dichotomy, then system* (6) *has an almost periodic solutionx*(*t*) *can be expressed as:*

$$\begin{aligned} x(t)=\int _{-\infty }^{t}X(t)PX^{-1}(\sigma (s))f(s)\Delta s-\int ^{+\infty }_{t}X(t)(I-P)X^{-1}(\sigma (s))f(s)\Delta s, \,\, t\in \mathbb {T}, \end{aligned}$$

*whereX*(*t*) *is the fundamental solution matrix of* (5).

## Positive almost periodic solutions for the Nicholson’s blowflies model

In this section, we will state and prove the sufficient conditions for the existence and exponential stability of positive almost periodic solutions of (2). Throughout this section, we restrict our discussion under Definition 7.

Set $\mathbb {B}=\{\varphi \in C(\mathbb {T},\mathbb {R}^n):\varphi =(\varphi _1,\varphi _2,\ldots ,\varphi _n)$ is an almost periodic function on $\mathbb {T}$} with the norm $||\varphi ||_\mathbb {B}=\sup \limits _{t\in \mathbb {T}}||\varphi (t)||$, where $||\varphi (t)||=\max \limits _{1\le i\le n}|\varphi _i(t)|$, then $\mathbb {B}$ is a Banach space. Denote $\mathbb {C}=C([t_0-\theta ,t_0]_\mathbb {T},\mathbb {R}^n)$ and $C\{A_1,A_2\}=\{\varphi =(\varphi _1,\varphi _2,\ldots ,\varphi _n)\in \mathbb {C} : A_1\le \varphi _i(s)\le A_2, s\in [t_0-\theta , t_0]_\mathbb {T},\,i=1,2,\ldots ,n\}$, where $0<A_1<A_2$ are constants.

In the proofs of our results of this section, we need the following facts: The function $xe^{-x}$ decreases on $[1, +\infty )$.

### **Lemma 9**

*Assume that the following conditions hold.*

$$(H_{1})$$
$$c_i, b_{ik}, \beta _{ij}, \alpha _{ij},\tau _{ij}\in AP(\mathbb {T},\mathbb {R}^+)$$ and $$c_i^->0, b_{ik}^->0, \beta _{ij}^->0, \alpha _{ij}^->0$$, $$t-\tau _{ij}(t)\in \mathbb {T}$$, $$i,k,j=1,2,\ldots ,n$$.
$$(H_{2})$$
$$\sum \nolimits _{k=1,k\ne i}^n\frac{b_{ik}^+}{c_{i}^-}<1, \quad i=1,2,\ldots ,n$$.
$$(H_{3})$$
*There exist positive constants*$A_1, A_2$*satisfy*
  $$\begin{aligned} A_2> \max \limits _{1\le i\le n}\left \{\left [1-\sum \limits _{k=1,k\ne i}^n\frac{b_{ik}^+}{c_{i}^-}\right ]^{-1}\sum \limits _{j=1}^n \frac{\beta _{ij}^+}{c_i^-\alpha _{ij}^-e}\right \} \end{aligned}$$
  *and*
  $$\begin{aligned} \min \limits _{1\le i\le n}\left \{\left [1-\sum \limits _{k=1,k\ne i}^n\frac{b_{ik}^-}{c_{i}^+}\right ]^{-1}\sum \limits _{j=1}^n A_2\frac{\beta _{ij}^-}{c_{i}^+}e^{-\alpha _{ij}^+A_2}\right \}>A_1\ge \frac{1}{\min \limits _{1\le i,j\le n}\{\alpha _{ij}^-\}}. \end{aligned}$$

*Then the solution*$x(t)=(x_1(t),x_2(t),\ldots ,x_n(t))$*of* (2) *with the initial value*$\varphi \in C\{A_1,A_2\}$*satisfies*

$$\begin{aligned} A_1<x_i(t)< A_2,\,\,t\in [t_0, +\infty )_\mathbb {T},\,\quad i=1,2,\ldots ,n. \end{aligned}$$

### *Proof*

Let $x(t)=x(t;t_0,\varphi )$, where $\varphi \in C\{A_1,A_2\}$. At first, we prove that

7 $$\begin{aligned} 0<x_i(t)<A_2,\,\,t\in [t_0, \eta (\varphi ))_\mathbb {T},\quad i=1,2,\ldots ,n, \end{aligned}$$

where $[t_0, \eta (\varphi ))_\mathbb {T}$ is the maximal right-interval of existence of $x(t;t_0,\varphi )$. By way of contradiction, assume that (7) does not hold. Then, there exists $i_0\in \{1,2,\dots ,n\}$ and the first time $t_1\in [t_0, \eta (\varphi ))_\mathbb {T}$ such that

$$\begin{aligned}&x_{i_0}(t_1)\ge A_2, \,\,x_{i_0}(t)<A_2,\,\, t\in [t_0-\theta ,t_1)_{\mathbb {T}},\\&\quad x_{k}(t)\le A_2,\,\, \mathrm {for} \, k\ne i_0, \,\,t\in [t_0-\theta ,t_1]_{\mathbb {T}},\quad k=1,2,\dots ,n. \end{aligned}$$

Therefore, there must be a positive constant $a\ge 1$ such that

$$\begin{aligned} x_{i_0}(t_1)&= aA_2, \,\,x_{i_0}(t)<aA_2,\,\, t\in [t_0-\theta ,t_1)_{\mathbb {T}},\\&\quad x_{k}(t)\le aA_2,\,\, \mathrm {for} \, k\ne i_0, \,\,t\in [t_0-\theta ,t_1]_{\mathbb {T}},\quad k=1,2,\dots ,n. \end{aligned}$$

In view of the fact that $\sup \limits _{u\ge 0}ue^{-u}=\frac{1}{e}$ and $a\ge 1$, we can obtain

$$\begin{aligned} 0\le x_{i_0}^{\Delta }(t_1)&= -c_{i_0}(t_1)x_{i_0}(t_1)+\sum \limits _{k=1,k\ne i_0}^nb_{i_0k}(t_1)x_k(t_1)\nonumber \\&\quad +\sum \limits _{j=1}^n \frac{\beta _{i_0j}(t_1)}{\alpha _{i_0j}(t_1)}\alpha _{i_0j}(t_1)x_{i_0}(t_1-\tau _{i_0j}(t_1)) e^{-\alpha _{i_0j}(t_0)x_{i_0}(t_0-\tau _{i_0j}(t_0))}\nonumber \\&\le -c_{i_0}^-aA_2+\sum \limits _{k=1,k\ne i_0}^nb_{i_0k}^+aA_2+\sum \limits _{j=1}^n \frac{\beta _{i_0j}^+}{\alpha _{i_0j}^-}\cdot \frac{1}{e}\nonumber \\&\le ac_{i_0}^-\left (-A_2+\sum \limits _{k=1,k\ne i_0}^n\frac{A_2b_{i_0k}^+}{c_{i_0}^-}+\sum \limits _{j=1}^n \frac{\beta _{i_0j}^+}{c_{i_0}^-\alpha _{i_0j}^-e}\right )<0, \end{aligned}$$

which is a contradiction and hence (7) holds. Next, we show that

8 $$\begin{aligned} x_i(t)> A_1,\,\,t\in [t_0, \eta (\varphi ))_{\mathbb{T}},\,\quad i=1,2,\ldots ,n. \end{aligned}$$

By way of contradiction, assume that (8) does not hold. Then, there exists $i_1\in \{1,2,\dots ,n\}$ and the first time $t_2\in [t_0, \eta (\varphi ))_\mathbb {T}$ such that

$$\begin{aligned}&x_{i_1}(t_2)\le A_1, \quad x_{i_1}(t)>A_1,\,\, t\in [t_0-\theta ,t_2)_{\mathbb {T}},\\&\quad x_{k}(t)\ge A_1,\,\, \mathrm {for} \, k\ne i_1, \quad t\in [t_0-\theta ,t_2]_{\mathbb {T}},\quad k=1,2,\dots ,n. \end{aligned}$$

Therefore, there must be a positive constant $c\le 1$ such that

$$\begin{aligned} x_{i_1}(t_2)&= cA_1, \,\,x_{i_1}(t)>cA_1,\quad t\in [t_0-\theta ,t_2)_{\mathbb {T}},\\&x_{k}(t)\ge cA_1,\,\, \mathrm {for} \, k\ne i_1, \,\,t\in [t_0-\theta ,t_2]_{\mathbb {T}},\quad k=1,2,\dots ,n. \end{aligned}$$

Noticing that $c\le 1$, it follows that

$$\begin{aligned} 0\ge x_{i_1}^{\Delta }(t_2)& = -c_{i_1}(t_2)x_{i_1}(t_2)+\sum \limits _{k=1,k\ne i_1}^nb_{i_1k}(t_2)x_k(t_2)\nonumber \\&\quad +\sum \limits _{j=1}^n \beta _{i_1j}(t_2)x_{i_1}(t_2-\tau _{i_1j}(t_2)) e^{-\alpha _{i_1j}(t_2)x_{i_1}(t_2-\tau _{i_1j}(t_2))} \\&\ge -c_{i_1}^+cA_1+\sum \limits _{k=1,k\ne i_1}^nb_{i_1k}^-cA_1+\sum \limits _{j=1}^n A_2\frac{\alpha _{i_1j}^+\beta _{i_1j}^-}{\alpha _{i_1j}^+}e^{-\alpha _{i_1j}^+A_2}\nonumber \\&= c c_{i_1}^+\left (-A_1+\sum \limits _{k=1,k\ne i_1}^nA_1\frac{b_{i_1k}^-}{c_{i_1}^+}+\sum \limits _{j=1}^n A_2\frac{\beta _{i_1j}^-}{c_{i_1}^+}e^{-\alpha _{i_1j}^+A_2}\right )>0, \end{aligned}$$

which is a contradiction and hence (8) holds. Similar to the proof of Theorem 2.3.1 in Hale and Verduyn Lunel (1993), we easily obtain $\eta (\varphi )=+\infty$. This completes the proof. $\square$

### *Remark 5*

If $\mathbb {T}=\mathbb {R}$, then $\mu (t)\equiv 0$, so, $-c_i\in \mathcal {R}^+$. If $\mathbb {T}=\mathbb {Z}$, then $\mu (t)\equiv 1$, so, $-c_i\in \mathcal {R}^+$ if and only if $c_i<1$.

### **Theorem 10**

*Assume that*$(H_1)$*and*$(H_3)$*hold. Suppose further that*

$$(H_4)$$
$-c_i\in \mathcal {R}^+$, *where*$\mathcal {R}^+$*denotes the set of positive regressive functions*, $i=1,2,\ldots ,n$.
$$(H_5)$$
$$\sum \nolimits _{k=1,k\ne i}^nb_{ik}^++ \sum \nolimits ^{n}_{j=1}\frac{\beta _{ij}^+}{e^2}<c_i^-, \quad i=1,2,\ldots ,n.$$

*Then system* (2) *has a positive almost periodic solution in the region*$\mathbb {B}^*=\{\varphi |\,\,\varphi \in \mathbb {B}, A_1 \le \varphi _i(t)\le A_2, t\in \mathbb {T},\quad i=1,2,\ldots ,n\}$.

### *Proof*

For any given $\varphi \in \mathbb {B}$, we consider the following almost periodic dynamic system:

9 $$\begin{aligned} x_i^\Delta (t)= & {} -c_i(t)x_i(t)+\sum \limits _{k=1,k\ne i}^nb_{ik}(t)\varphi _k(t)\nonumber \\&+\sum \limits _{j=1}^n\beta _{ij}(t)\varphi _i(t-\tau _{ij}(t))e^{-\alpha _{ij}(t)\varphi _i(t-\tau _{ij}(t))},\quad i=1,2,\ldots ,n. \end{aligned}$$

Since $\min _{1\le i\le n}\{ c_i^-\}>0$, $t\in \mathbb {T}$, it follows from Lemma 3 that the linear system

$$\begin{aligned} x_i^\Delta (t)=-c_i(t)x_i(t),\,\,\quad i=1,2,\ldots ,n \end{aligned}$$

admits an exponential dichotomy on $\mathbb {T}$. Thus, by Lemma 8, we obtain that system (9) has an almost periodic solution $x_\varphi =(x_{\varphi _1},x_{\varphi _2},\ldots ,x_{\varphi _n})$, where

$$\begin{aligned} {x_{\varphi }}_i(t)& = \int _{-\infty }^te_{-c_i}(t,\sigma (s))\left [\sum \limits _{k=1,k\ne i}^nb_{ik}(s)\varphi _k(s)\right. \\&\quad\left. +\sum \limits _{j=1}^n\beta _{ij}(s)\varphi _i(s-\tau _{ij}(s))e^{-\alpha _{ij}(s)\varphi _i(s-\tau _{ij}(s))}\right ]\Delta s,\quad i=1,2,\ldots ,n. \end{aligned}$$

Define a mapping $T:\mathbb {B}^*\rightarrow \mathbb {B}^*$ by

$$\begin{aligned} T\varphi (t)=x_{\varphi }(t),\,\,\forall \varphi \in \mathbb {B}^*. \end{aligned}$$

Obviously, $\mathbb {B}^*=\{\varphi |\,\,\varphi \in \mathbb {B}, A_1 \le \varphi _i(t)\le A_2, t\in \mathbb {T}, \quad i=1,2,\ldots ,n\}$ is a closed subset of $\mathbb {B}$. For any $\varphi \in \mathbb {B}^*$, by use of $(H_2)$, we have

$$\begin{aligned} {x_{\varphi }}_i(t)& \le \int _{-\infty }^te_{-c_i^-}(t,\sigma (s))\bigg [\sum \limits _{k=1,k\ne i}^nb_{ik}^+A_2+\sum \limits _{j=1}^n\frac{\beta _{ij}^+}{\alpha _{ij}^-}\times \frac{1}{e}\bigg ]\Delta s\\&\le \frac{1}{c_i^-}\bigg [\sum \limits _{k=1,k\ne i}^nb_{ik}^+A_2+\sum \limits _{j=1}^n\frac{\beta _{ij}^+}{\alpha _{ij}^-}\times \frac{1}{e}\bigg ]\\&\le A_2,\,\,\quad i=1,2,\ldots ,n \end{aligned}$$

and we also have

$$\begin{aligned} {x_{\varphi }}_i(t)& \ge \int _{-\infty }^te_{-c_i^+}(t,\sigma (s))\bigg [\sum \limits _{k=1,k\ne i}^nA_1b_{ik}^-+\sum \limits _{j=1}^n {\beta _{ij}^-} \varphi _i(s-\tau _{ij}(s))e^{-\alpha _{ij}^+\varphi _i(s-\tau _{ij}(s))}\bigg ]\Delta s\\&\ge \frac{1}{c_i^+}\bigg [\sum \limits _{k=1,k\ne i}^nA_1b_{ik}^-+\sum \limits _{j=1}^nA_2\beta _{ij}^-e^{-\alpha _{ij}^+A_2}\bigg ]\\&\ge A_1,\,\,\quad i=1,2,\ldots ,n. \end{aligned}$$

Therefore, the mapping *T* is a self-mapping from $\mathbb {B}^*$ to $\mathbb {B}^*$.

Next, we prove that the mapping *T* is a contraction mapping on $\mathbb {B}^*$. Since $\sup \limits _{u\ge 1}|\frac{1-u}{e^u}|=\frac{1}{e^2}$, we find that

$$\begin{aligned} |xe^{-x}-ye^{-y}|&= \Big |\frac{1-(x+\xi (y-x))}{e^{x+\xi (y-x)}}\Big ||x-y|\\& \le \frac{1}{e^2}|x-y|,\,x,y\ge 1,\,0<\xi <1. \end{aligned}$$

For any $\varphi =(\varphi _1, \varphi _2, \ldots , \varphi _n)^T$, $\psi =(\psi _1, \psi _2, \ldots , \psi _n)^T \in \mathbb {B}^*$, we obtain that

$$\begin{aligned}&|(T\varphi )_i(t)-(T\psi )_i(t)|\nonumber \\&\quad \le \Big |\int _{-\infty }^t\mathrm {e}_{-c_i}(t,\sigma (s))\sum \limits _{k=1,k\ne i}^nb_{ik}(s)\big (\varphi _k(s)-\psi _k(s)\big )\Delta s\Big |\nonumber \\&\qquad+\bigg |\int _{-\infty }^t\mathrm {e}_{-c_i}(t,\sigma (s)) \sum \limits ^{n}_{j=1}\beta _{ij}(s)\bigg (\varphi _i(s-\tau _{ij}(s))e^{-\alpha _{ij}(s)\varphi _i(s-\tau _{ij}(s))} \nonumber \\&\quad\quad -\psi _i(s-\tau _{ij}(s))e^{-\alpha _{ij}(s)\psi _i(s-\tau _{ij}(s))}\bigg )\Delta s\bigg |\nonumber \\&\quad \le \frac{1}{c_i^-}\sum \limits _{k=1,k\ne i}^nb_{ik}^+\Vert \varphi -\psi \Vert _{\mathbb {B}}+\bigg |\int _{-\infty }^t\mathrm {e}_{-c_i}(t,\sigma (s)) \sum \limits ^{n}_{j=1}\frac{\beta _{ij}(s)}{\alpha _{ij}(s)}\Big (\alpha _{ij}(s)\varphi _i(s-\tau _{ij}(s))\nonumber \\&\quad \quad\times e^{-\alpha _{ij}(s)\varphi _i(s-\tau _{ij}(s))} -\alpha _{ij}(s)\psi _i(s-\tau _{ij}(s))e^{-\alpha _{ij}(s)\psi _i(s-\tau _{ij}(s))}\Big )\Delta s\bigg |\nonumber \\&\quad \le \bigg (\frac{1}{c_i^-}\sum \limits _{k=1,k\ne i}^nb_{ik}^++ \sum \limits ^{n}_{j=1}\frac{\beta _{ij}^+}{c_i^-e^2}\bigg )\Vert \varphi -\psi \Vert _{\mathbb {B}},\quad i=1,2,\ldots ,n. \end{aligned}$$

It follows that

$$\begin{aligned} \Vert T\phi -T\psi \Vert _\mathbb {B}<\max \limits _{1\le i\le n}\left \{\frac{1}{c_i^-}\sum \limits _{k=1,k\ne i}^nb_{ik}^++ \sum \limits ^{n}_{j=1}\frac{\beta _{ij}^+}{c_i^-e^2}\right \}\Vert \varphi -\psi \Vert _{\mathbb {B}}, \end{aligned}$$

which implies that *T* is a contraction. By the fixed point theorem in Banach space, *T* has a unique fixed point $\varphi ^*\in \mathbb {B}^*$ such that $T\varphi ^*=\varphi ^*$. In view of (9), we see that $\varphi ^*$ is a solution of (2). Therefore, (2) has a positive almost periodic solution in the region $\mathbb {B}^*$. This completes the proof. $\square$

### **Definition 8**

Let $x^*(t)=(x_1^*(t), x_2^*(t), \ldots , x_n^*(t))^T$ be an almost periodic solution of (2) with initial value $\varphi ^*(s)=(\varphi _1^*(s), \varphi _2^*(s), \ldots , \varphi _n^*(s))^T\in C\{A_1,A_2\}$. If there exist positive constants $\lambda$ with $\ominus \lambda \in \mathcal {R}^{+}$ and $M>1$ such that such that for an arbitrary solution $x(t)=(x_1(t), x_2(t), \ldots , x_n(t))^T$ of (2) with initial value $\varphi (s)=(\varphi _1(s), \varphi _2(s), \ldots , \varphi _n(s))^T\in C\{A_1,A_2\}$ satisfies

$$\begin{aligned} ||x(t)-x^*(t)||\le M||\varphi -\varphi ^*||_{\infty } e_{\ominus \lambda }(t,t_0),\,\, t_0\in [-\theta ,\infty )_{\mathbb {T}},\,t\ge t_0, \end{aligned}$$

where $||\varphi -\varphi ^*||_{\infty }=\max \limits _{1\le i\le n}\bigg \{\sup \limits _{t\in [t_0-\theta ,t_0]}|\varphi _i(t)-\varphi _i^*(t)|\bigg \}$ for $\varphi ,\psi \in C\{A_1,A_2\}$. Then the solution $x^*(t)$ is said to be exponentially stable.

### **Theorem 11**

Assume that $(H_1)$, $(H_3)$–$(H_5)$ hold. Then the positive almost periodic solution $x^*(t)$ in the region $\mathbb {B}^*$ of (2) is unique and exponentially stable.

### *Proof*

By Theorem 10, (2) has a positive almost periodic solution $x_i^*(t)$ in the region $\mathbb {B}^*$. Let $x(t)=(x_1(t),x_2(t),\ldots , x_n(t))^T$ be any arbitrary solution of (2) with initial value $\varphi (s)=(\varphi _1(s),\varphi _2(s),\ldots ,\varphi _n(s))^T\in C\{A_1,A_2\}$. Then it follows from (2) that for $t\ge t_0, i=1,2,\ldots ,n$,

10 $$\begin{aligned} (x_i(t)-x_i^*(t))^\Delta \nonumber & =-c_i(t)(x_i(t)-x_i^*(t))+\sum \limits _{k=1,k\ne i}^nb_{ik}(t)(x_k(t)-x_k^*(t))\nonumber \\& \quad+\sum \limits _{j=1}^n\beta _{ij}(t)\big [x_i(t-\tau _{ij}(t))e^{-\alpha _{ij}(t)x_i(t-\tau _{ij}(t))} -x_i^*(t-\tau _{ij}(t))e^{-\alpha _{ij}(t)x_i^*(t-\tau _{ij}(t))}\big ]. \end{aligned}$$

The initial condition of (10) is

$$\begin{aligned} \psi _i(s)=\varphi _i(s)-x_i^*(s),\,\,s\in [t_0-\theta , t_0]_{\mathbb {T}},\,\quad i=1,2,\ldots ,n. \end{aligned}$$

For convenience, we denote $u_i(t)=x_i(t)-x_i^*(t), \quad i=1,2,\ldots ,n$. Then, by (10), we have

11 $$\begin{aligned} u_i(t)& = u_i(t_0)e_{-c_i}(t,t_0)+\int _{t_0}^{t}e_{-c_{i}}(t,\sigma (s))\sum \limits _{k=1,k\ne i}^nb_{ik}(s)u_k(s)\Delta s\nonumber \\&\quad +\int _{t_0}^{t}e_{-c_{i}}(t,\sigma (s))\sum \limits _{j=1}^n\beta _{ij}(s)\bigg [x_i(s-\tau _{ij}(s))e^{-\alpha _{ij}(s)x_i(s-\tau _{ij}(s))} \nonumber \\&\quad -x_i^*(s-\tau _{ij}(s))e^{-\alpha _{ij}(s)x_i^*(s-\tau _{ij}(s))}\bigg ]\Delta s, \,\,t\ge t_0, \quad i=1,2,\ldots ,n. \end{aligned}$$

For $\omega \in \mathbb {R}$, let $\Gamma _{i}(\omega )$ be defined by

$$\begin{aligned} \Gamma _{i}(\omega )= & {} c_{i}^{-}-\omega -\exp \{\omega \sup \limits _{s\in \mathbb {T}}\mu (s)\}\bigg (\sum \limits _{k=1,k\ne i}^nb_{{i}k}^++\frac{1}{e^2}\sum \limits ^{n}_{j=1}\beta _{ij}^+\exp \{\omega \tau _{ij}^+\}\bigg ),\quad i=1,2,\ldots ,n. \end{aligned}$$

In view of $(H_{2})$, we have that

$$\begin{aligned} \Gamma _{i}(0)=c_{i}^{-}-\bigg (\sum \limits _{k=1,k\ne i}^nb_{{i}k}^++\frac{1}{e^2}\sum \limits ^{n}_{j=1}\beta _{ij}^+\bigg )> 0, \quad i=1,2,\ldots ,n. \end{aligned}$$

Since $\Gamma _{i}(\omega )$ is continuous on $[0,+\infty )$ and $\Gamma _{i}(\omega )\rightarrow -\infty$ as $\omega \rightarrow +\infty$, so there exists $\omega _{i}> 0$ such that $\Gamma _{i}(\omega _{i})=0$ and $\Gamma _{i}(\omega )> 0$ for $\omega \in (0,\omega _{i}), \quad i=1,2,\ldots ,n$. By choosing a positive constant $a=\min \big \{\omega _{1},\omega _{2},\ldots ,\omega _{n}\big \}$, we have $\Gamma _{i}(a)\ge 0, \quad i=1,2,\ldots ,n.$ Hence, we can choose a positive constant $0< \alpha < \min \big \{a,\min \limits _{1\le i \le n}\{c_{i}^{-}\}\big \}$ such that

$$\begin{aligned} \Gamma _{i}(\alpha )>0,\,\,\quad i=1,2,\ldots ,n, \end{aligned}$$

which implies that

$$\begin{aligned}&\frac{\exp \{\alpha \sup \limits _{s\in \mathbb {T}}\mu (s)\}}{c_{i}^{-}-\alpha }\left (\sum \limits _{k=1,k\ne i}^nb_{{i}k}^++\frac{1}{e^2}\sum \limits ^{n}_{j=1}\beta _{ij}^+\exp \{\alpha \tau _{ij}^+\}\right )< 1,\quad i=1,2,\ldots ,n. \end{aligned}$$

Take

$$\begin{aligned} M=\max \limits _{1\le i\le n}\left \{\frac{c_{i}^-}{\sum \nolimits _{k=1,k\ne i}^nb_{{i}k}^++\frac{1}{e^2}\sum \nolimits ^{n}_{j=1}\beta _{ij}^+}\right \}. \end{aligned}$$

It follows from $(H_5)$ that $M>1$. Besides, we can obtain that

$$\begin{aligned} \frac{1}{M}< & {} \frac{\exp \{\alpha \sup \limits _{s\in \mathbb {T}}\mu (s)\}}{c_{i}^{-}-\alpha }\left (\sum \nolimits _{k=1,k\ne i}^nb_{{i}k}^++\frac{1}{e^2}\sum \nolimits ^{n}_{j=1}\beta _{ij}^+\exp \{\alpha \tau _{ij}^+\right ). \end{aligned}$$

In addition, noticing that $e_{\ominus \alpha }(t,t_0)\ge 1$ for $t\in [t_0-\theta ,t_0]_{\mathbb {T}}$. Hence, it is obvious that

$$\begin{aligned} ||u(t)||\le M e_{\ominus \alpha }(t,t_0)\Vert \psi \Vert _{\infty },\quad \forall \, t\in [t_0-\theta ,t_0]_{\mathbb {T}}. \end{aligned}$$

We claim that

12 $$\begin{aligned} ||u(t)||\le M e_{\ominus \alpha }(t,t_0)\Vert \psi \Vert _{\infty },\,\,\,\,\,\forall \, t\in (t_0,+\infty )_{\mathbb {T}}. \end{aligned}$$

To prove this claim, we show that for any $p>1$, the following inequality holds

13 $$\begin{aligned} ||u(t)||< pM e_{\ominus \alpha }(t,t_0)\Vert \psi \Vert _{\infty },\,\,\,\,\,\forall \, t\in (t_0,+\infty )_{\mathbb {T}}, \end{aligned}$$

which implies that, for $i=1,2,\ldots ,n$, we have

14 $$\begin{aligned} |u_i(t)|< pM e_{\ominus \alpha }(t,t_0)\Vert \psi \Vert _{\infty },\quad \forall t\in (t_0,+\infty )_{\mathbb {T}}. \end{aligned}$$

By way of contradiction, assume that (14) is not true. Then there exists $t_1\in (t_0,+\infty )_{\mathbb {T}}$ and $i_0\in \{1,2,\ldots ,n\}$ such that

$$\begin{aligned}&|u_{i_0}(t_1)|\ge pM e_{\ominus \alpha }(t_1,t_0)\Vert \psi \Vert _{\infty }, \,\,|u_{i_0}(t)|<pM e_{\ominus \alpha }(t,t_0)\Vert \psi \Vert _{\infty },\,\, t\in (t_0,t_1)_{\mathbb {T}},\\&\quad |u_{k}(t)|\le pM e_{\ominus \alpha }(t,t_0)\Vert \psi \Vert _{\infty },\,\,\mathrm {for} \, k\ne i_0,\, \,t\in (t_0,t_1]_{\mathbb {T}},\quad k=1,2,\dots ,n. \end{aligned}$$

Therefore, there must be a constant $q\ge 1$ such that

$$\begin{aligned} |u_{i_0}(t_1)|&= qpM e_{\ominus \alpha }(t_1,t_0)\Vert \psi \Vert _{\infty }, \,\,|u_{i_0}(t)|<qpM e_{\ominus \alpha }(t,t_0)\Vert \psi \Vert _{\infty },\,\, t\in (t_0,t_1)_{\mathbb {T}},\\&|u_{k}(t)|<qpM e_{\ominus \alpha }(t_1,t_0)\Vert \psi \Vert _{\infty },\,\,\mathrm {for} \, k\ne i_0,\, \,t\in (t_0,t_1]_{\mathbb {T}},\quad k=1,2,\dots ,n. \end{aligned}$$

According to (11), we have

$$\begin{aligned} |u_{i_0}(t_1)|&= \bigg |u_{i_0}(t_0)e_{-c_{i_0}}(t_1,t_0)+\int _{t_0}^{t_1}e_{-c_{{i_0}}}(t_1,\sigma (s))\sum \limits _{k=1,k\ne i_0}^nb_{{i_0}k}(s)u_k(s)\Delta s\\&+\int _{t_0}^{t_1}e_{-c_{{i_0}}}(t_1,\sigma (s)) \sum \limits _{j=1}^n\beta _{{i_0}j}(s)\big [x_{i_0}(s-\tau _{{i_0}j}(s))e^{-\alpha _{{i_0}j}(s)x_{i_0}(s-\tau _{{i_0}j}(s))}\\&-x_{i_0}^*(s-\tau _{{i_0}j}(s))e^{-\alpha _{{i_0}j}(s)x_{i_0}^*(s-\tau _{{i_0}j}(s))}\big ]\Delta s\bigg |\\&\le e_{-c_{i_0}}(t_1,t_0)\Vert \psi \Vert _{\infty }+qpM e_{\ominus \alpha }(t_1,t_0)\Vert \psi \Vert _{\infty }\\&\times \int _{t_0}^{t_1}e_{-c_{i_0}}(t_1,\sigma (s))e_{\alpha }(t_1,\sigma (s)) \bigg (\sum \limits _{k=1,k\ne i_0}^nb_{{i_0}k}^+e_{\alpha }(\sigma (s),s)\\&+\sum \limits ^{m}_{j=1}\frac{\beta _{i_0j}^+}{e^2}e_{\alpha }(\sigma (s),s-\tau _{i_0j}(s))\bigg )\Delta s\\&\le e_{-c_{i_0}}(t_1,t_0)\Vert \psi \Vert _{\infty }+qpM e_{\ominus \alpha }(t_1,t_0)\Vert \psi \Vert _{\infty }\\&\times \int _{t_0}^{t_1}e_{-c_{i_0}\oplus \alpha }(t_1,\sigma (s)) \bigg (\sum \limits _{k=1,k\ne i}^nb_{{i_0}k}^+\exp \{\alpha \sup \limits _{s\in \mathbb {T}}\mu (s)\}\\&+\sum \limits ^{m}_{j=1}\frac{\beta _{i_0j}^+}{e^2}\exp \{\alpha (\tau _{i_0j}^++\sup \limits _{s\in \mathbb {T}}\mu (s))\}\bigg )\Delta s\\&= e_{-c_{i_0}}(t_1,t_0)\Vert \psi \Vert _{\infty }+qpM e_{\ominus \alpha }(t_1,t_0)\Vert \psi \Vert _{\infty }\exp \{\alpha \sup \limits _{s\in \mathbb {T}}\mu (s)\}\bigg (\sum \limits _{k=1,k\ne i_0}^nb_{{i_0}k}^+\\&+ \sum \limits ^{m}_{j=1}\frac{\beta _{i_0j}^+}{e^2}\exp \{\alpha \tau _{i_0j}^+\}\bigg ) \int _{t_0}^{t_1}e_{-c_{i_0}\oplus \alpha }(t_1,\sigma (s)) \Delta s \nonumber \\&= qpM e_{\ominus \alpha }(t_1,t_0)\Vert \psi \Vert _{\infty }\bigg \{\frac{1}{qpM}e_{-c_{i_0}\oplus \alpha }(t_1,t_0) +\exp \{\alpha \sup \limits _{s\in \mathbb {T}}\mu (s)\}\bigg (\sum \limits _{k=1,k\ne i_0}^nb_{{i_0}k}^+\nonumber \\&+ \sum \limits ^{m}_{j=1}\frac{\beta _{i_0j}^+}{e^2}\exp \{\alpha \tau _{i_0j}^+\}\bigg ) \int _{t_0}^{t_1}e_{-c_{i_0}\oplus \alpha }(t_1,\sigma (s))\Delta s\bigg \} \nonumber \\&<qpM e_{\ominus \alpha }(t_1,t_0)\Vert \psi \Vert _{\infty }\bigg \{\frac{1}{qpM}e_{-(c_{i_0}^{-}-\alpha )}(t_1,t_0) +\exp \{\alpha \sup \limits _{s\in \mathbb {T}}\mu (s)\}\bigg (\sum \limits _{k=1,k\ne i_0}^nb_{{i_0}k}^+\nonumber \\&+ \sum \limits ^{m}_{j=1}\frac{\beta _{i_0j}^+}{e^2}\exp \{\alpha \tau _{i_0j}^+\}\bigg ) \frac{1}{-(c_{i_0}^{-}-\alpha )}\int _{t_0}^{t_1}\big (-(c_{i_0}^{-}-\alpha )\big )e_{-(c_{i_0}^{-}-\alpha )}(t_1,\sigma (s))\Delta s\bigg \} \\\le & {} qpM e_{\ominus \alpha }(t_1,t_0)\Vert \psi \Vert _{\infty }\Bigg \{\bigg [\frac{1}{qpM}-\frac{\exp \{\alpha \sup \limits _{s\in \mathbb {T}}\mu (s)\}}{c_{i_0}^{-}-\alpha }\Big (\sum \limits _{k=1,k\ne i_0}^nb_{{i_0}k}^+\\&+\frac{1}{e^2}\sum \limits ^{n}_{j=1}\beta _{ij}^+ \exp \{\alpha \tau _{i_0j}^+\}\Big )\bigg ]e_{-(c_{i_0}^{-}-\alpha )}(t_1,t_0) +\frac{\exp \{\alpha \sup \limits _{s\in \mathbb {T}}\mu (s)\}}{c_{i_0}^{-}-\alpha }\Big (\sum \limits _{k=1,k\ne i_0}^nb_{{i_0}k}^+\\&+\frac{1}{e^2}\sum \limits ^{n}_{j=1}\beta _{i_0j}^+ \exp \{\alpha \tau _{i_0j}^+\}\Big )\Bigg \} \\<&qpM e_{\ominus \alpha }(t_1,t_0)\Vert \psi \Vert _{\infty }\Bigg \{\bigg [\frac{1}{M}-\frac{\exp \{\alpha \sup \limits _{s\in \mathbb {T}}\mu (s)\}}{c_{i_0}^{-}-\alpha }\Big (\sum \limits _{k=1,k\ne i_0}^nb_{{i_0}k}^+\\&+\frac{1}{e^2}\sum \limits ^{n}_{j=1}\beta _{i_0j}^+ \exp \{\alpha \tau _{i_0j}^+\}\Big )\bigg ]e_{-(c_{i_0}^{-}-\alpha )}(t_1,t_0) +\frac{\exp \{\alpha \sup \limits _{s\in \mathbb {T}}\mu (s)\}}{c_{i_0}^{-}-\alpha }\Big (\sum \limits _{k=1,k\ne i_0}^nb_{{i_0}k}^+\\&+\frac{1}{e^2}\sum \limits ^{n}_{j=1}\beta _{i_0j}^+ \exp \{\alpha \tau _{i_0j}^+\}\Big )\Bigg \} \\< & {} qpM e_{\ominus \alpha }(t_1,t_0)\Vert \psi \Vert _{\infty }, \end{aligned}$$

which is a contradiction. Therefore, (14) and (13) hold. Let $p\rightarrow 1$, then (12) holds. Hence, we have that

$$\begin{aligned} ||u(t)||\le M\Vert \psi \Vert _{\infty }e_{\ominus \alpha }(t,t_0),\,\, t\in [t_0,+\infty )_{\mathbb {T}}, \end{aligned}$$

which implies that the positive almost periodic solution $x^*(t)$ of (2) is exponentially stable. The exponential stability of $x^*(t)$ implies that the uniqueness of the positive almost periodic solution. The proof is complete. $\square$

### *Remark 6*

It is easy to see that under definitions of almost periodic time scales and almost periodic functions in Li and Wang (2011a), the conclusions of Theorems 10 and 11 are true.

### *Remark 7*

From Remark 5, Theorems 10 and 11, we can easily see that if $c_i(t)<1, i=1,2,\ldots ,n$, then the continuous-time Nicholson’s blowflies models and the discrete-time analogue have the same dynamical behaviors. This fact provides a theoretical basis for the numerical simulation of continuous-time Nicholson’s blowflies models.

### *Remark 8*

Our results and methods of this paper are different from those in Li and Yang (2012).

### *Remark 9*

When $\mathbb {T}=\mathbb {R}$ or $\mathbb {T}=\mathbb {Z}$, our results of this section are also new. If we take $\mathbb {T}=\mathbb {R}, A_1=1, A_2=e$, then Lemma 9, Theorems 10 and 11 improve Lemma 2.4, Theorems 2 and 3 in Wang et al. (2011), respectively.

## An example

In this section, we present an example to illustrate the feasibility of our results obtained in previous sections.

### *Example 3*

In system (2), let $n=3$ and take coefficients as follows:

$$\begin{aligned} c_1(t)& = 0.21+0.01\sin \sqrt{3}t,\,\,c_2(t)=0.24+0.008|\sin 2t|,\,\,c_3(t)=0.41+0.01\cos \sqrt{2}t,\\ b_{12}(t)& = 0.04-0.001|\cos \pi t|,\,\,b_{13}(t)=0.07-0.002|\cos \sqrt{3} t|,\,\,b_{21}(t)=0.06-0.002|\cos \sqrt{3} t|,\\ b_{23}(t)& = 0.06-0.001|\sin \sqrt{2} t|,\,\, b_{31}(t)=0.17-0.01|\sin \sqrt{3}t|,\,\,b_{32}(t)=0.14-0.01|\cos \sqrt{2} t|,\\ \beta _{11}(t)& = 0.09-0.01|\sin \pi t|,\,\,\beta _{12}(t)=0.16-0.01|\cos \sqrt{3}t|,\,\,\beta _{13}(t)=0.16-0.01|\sin t|,\\ \beta _{21}(t)& = 0.15-0.001|\cos \pi t|,\, \beta _{22}(t)=0.19-0.009|\cos \sqrt{3}t|,\,\beta _{23}(t)=0.09+0.01|\cos t|,\\ \beta _{31}(t)& = 0.16-0.002|\cos t|,\, \beta _{32}(t)=0.13-0.001|\cos \sqrt{2}t|,\,\,\beta _{33}(t)=0.11-0.008|\sin t|,\\ \alpha _{11}(t)& = \alpha _{12}(t)=\alpha _{13}(t)=0.999+0.001|\sin \sqrt{3}t|,\\ \alpha _{21}(t)& = 0.998+0.002\sin \sqrt{2}t,\,\, \alpha _{22}(t)=0.998+0.002\cos \sqrt{2}t,\,\, \alpha _{23}(t)=0.998+0.002\sin \pi t,\\ \alpha _{31}(t)& = 0.998+0.002|\sin t|,\,\, \alpha _{32}(t)=0.998+0.002|\sin \sqrt{3}t|,\,\, \alpha _{33}(t)=0.998+0.002\big |\sin \big (\frac{4}{3}t\big )\big |,\\ \tau _{11}(t)& = e^{0.2|\sin \pi t|},\,\,\tau _{12}(t)=e^{0.4|\cos (\pi t+\frac{\pi }{2}) |},\,\,\tau _{13}(t)=e^{0.5|\sin \pi t|},\\ \tau _{21}(t)& = e^{0.2|\cos (\pi t+\frac{\pi }{2}) |},\,\,\tau _{22}(t)=e^{0.3|\sin 3\pi t |},\,\,\tau _{23}(t)=e^{0.4|\cos 2\pi t|},\\ \tau _{31}(t)& = e^{0.5|\sin (\pi t+\frac{3\pi }{2}) |},\,\,\tau _{32}(t)=e^{0.3|\cos (\pi t+\frac{\pi }{2}) |},\,\,\tau _{33}(t)=e^{0.5|\cos 3\pi t|}. \end{aligned}$$

By calculating, we have

$$\begin{aligned} c_1^-& = 0.2,\,\, c_1^+=0.22,\,\,c_{2}^{-}=0.24,\,\,c_{2}^{+}=0.248,\,\,c_{3}^{-}=0.4,\,\,c_{3}^{+}=0.43,\\ b_{12}^-& = 0.039,\,\, b_{12}^+=0.04, \,\,b_{13}^-=0.068,\,\, b_{13}^+=0.07,\,\, b_{21}^-=0.058,\,\, b_{21}^+=0.06,\\ b_{23}^-& = 0.059,\,\, b_{23}^+=0.06,\,\, b_{31}^-=0.16,\,\, b_{31}^+=0.17,\,\, b_{32}^-=0.13,\,\, b_{32}^+=0.14,\\ \beta _{11}^-& = 0.08,\,\, \beta _{11}^+=0.09,\,\, \beta _{12}^-=0.15,\,\, \beta _{12}^+=0.16,\,\, \beta _{13}^-=0.15,\,\, \beta _{13}^+=0.16,\\ \beta _{21}^-& = 0.149, \,\,\beta _{21}^+=0.15, \,\,\beta _{22}^-=0.181,\,\, \beta _{22}^+=0.19,\,\, \beta _{23}^-=0.09, \,\,\beta _{23}^+=0.1,\\ \beta _{31}^-& = 0.158,\,\, \beta _{31}^+=0.16, \,\,\beta _{32}^-=0.129,\,\, \beta _{32}^+=0.13,\,\, \beta _{33}^-=0.103, \,\,\beta _{33}^+=0.11,\\ \alpha _{11}^-& = \alpha _{12}^-=\alpha _{13}^-=0.999,\,\,\alpha _{11}^+=\alpha _{12}^+=\alpha _{13}^+=1,\,\, \alpha _{21}^-=\alpha _{22}^-=\alpha _{23}^-=0.996,\\ \alpha _{21}^+& = \alpha _{22}^+=\alpha _{23}^+=1,\,\,\alpha _{31}^-=\alpha _{32}^-=\alpha _{33}^-=0.998,\,\, \alpha _{31}^+=\alpha _{32}^+=\alpha _{33}^+=1. \end{aligned}$$

Hence

$$\begin{aligned}&\sum \limits _{k=1,k\ne 1}^3\frac{b_{1k}^+}{c_{1}^-}=\frac{b_{12}^+}{c_{1}^-}+\frac{b_{13}^+}{c_{1}^-}=\frac{0.04+0.07}{0.2}=\frac{11}{20}<1,\\&\quad \sum \limits _{k=1,k\ne 2}^3\frac{b_{2k}^+}{c_{2}^-}=\frac{b_{21}^+}{c_{2}^-}+\frac{b_{23}^+}{c_{2}^-}=\frac{0.06+0.06}{0.24}=\frac{1}{2}<1,\\&\quad \sum \limits _{k=1,k\ne 3}^3\frac{b_{3k}^+}{c_{3}^-}=\frac{b_{31}^+}{c_{3}^-}+\frac{b_{32}^+}{c_{3}^-}=\frac{0.17+0.14}{0.4}= \frac{31}{40}<1,\\&\quad b_{12}^++b_{13}^++\frac{\beta _{11}^+}{e^2}+\frac{\beta _{12}^+}{e^2}+\frac{\beta _{13}^+}{e^2} =0.04+0.07+\frac{0.09}{e^2}+\frac{0.16}{e^2}+\frac{0.16}{e^2}\approx 0.165<c_{1}^-=0.2,\\&\quad b_{21}^++b_{23}^++\frac{\beta _{21}^+}{e^2}+\frac{\beta _{22}^+}{e^2}+\frac{\beta _{23}^+}{e^2} =0.06+0.06+\frac{0.15}{e^2}+\frac{0.19}{e^2}+\frac{0.1}{e^2}\approx 0.1795<c_{2}^-=0.24,\\&\quad b_{31}^++b_{32}^++\frac{\beta _{31}^+}{e^2}+\frac{\beta _{32}^+}{e^2}+\frac{\beta _{33}^+}{e^2} =0.17+0.14+\frac{0.16}{e^2}+\frac{0.13}{e^2}+\frac{0.11}{e^2}\approx 0.364<c_{3}^-=0.4. \end{aligned}$$

We can check that for any $A_{2}=1.68$, we have

$$\begin{aligned} A_2>\max \limits _{1\le i\le 3}\bigg \{\bigg [1-\sum \limits _{k=1,k\ne i}^3\frac{b_{ik}^+}{c_{i}^-}\bigg ]^{-1}\sum \limits _{j=1}^3 \frac{\beta _{ij}^+}{c_i^-\alpha _{ij}^-e}\bigg \}=\max \limits _{1\le i\le 3}\{1.678,1.354, 1.638\}=1.678. \end{aligned}$$

and

$$\begin{aligned}&\min \limits _{1\le i\le 3}\bigg \{\bigg [1-\sum \limits _{k=1,k\ne i}^3\frac{b_{ik}^-}{c_{i}^+}\bigg ]^{-1}\sum \limits _{j=1}^3 A_2\frac{\beta _{ij}^-}{c_{i}^+}e^{-\alpha _{ij}^+A_2}\bigg \}\\&\quad =\min \limits _{1\le i\le 3}\{1.053,1.025, 1.104\}\\&\quad =1.025>A_1>\frac{1}{\min \limits _{1\le i,j\le 3}\alpha _{ij}^-}=\frac{1}{0.996}\approx 1.004. \end{aligned}$$

For $0\le \mu (t)\le 1$, we have $1-\mu (t)c_{i}(t)>0$, therefore, whether $\mathbb {T}=\mathbb {R}$ or $\mathbb {T}=\mathbb {Z}$, we have $-c_{i}\in \mathcal {R}^{+}$ and condition $(H_{4})$ is satisfied.

If $-c_i\in \mathcal {R}^+$, that is, $1-c_i(t)\mu (t)>0, i=1,2,3$, then it is easy to verify that all conditions of Theorem 11 are satisfied. Therefore, the system in Example 4.1 has a unique positive almost periodic solution in the region $\mathbb {B}^*=\{\varphi |\,\,\varphi \in \mathbb {B}, 1.004<A_1\le \varphi _i(t)\le 1.68, t\in \mathbb {T}, \quad i=1,2,\ldots ,n\}$, which is exponentially stable.

Especially, if we take $\mathbb {T}=\mathbb {R}$ or $\mathbb {T}=\mathbb {Z}$, then $1-c_i(t)\mu (t)>0, i=1,2,3$. Hence, in this case, the continuous-time Nicholson’s blowflies model (2) and its discrete-time analogue have the same dynamical behaviors (see Figs. 1,2, 3, 4, 5, 6, 7, 8).

**Fig. 1:**
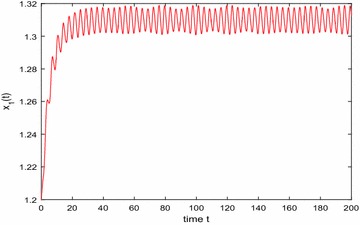
$\mathbb {T}=\mathbb {R}.$ Numerical solution $x_1(t)$ of system (7) for $(\varphi _1(0),\varphi _2(0),\varphi _3(0))=(1.2,1.25,1.2)$

**Fig. 2:**
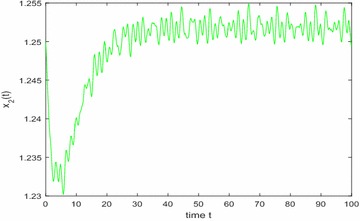
$\mathbb {T}=\mathbb {R}.$ Numerical solution $x_2(t)$ of system (7) for $(\varphi _1(0),\varphi _2(0),\varphi _3(0))=(1.2,1.25,1.2)$

**Fig. 3:**
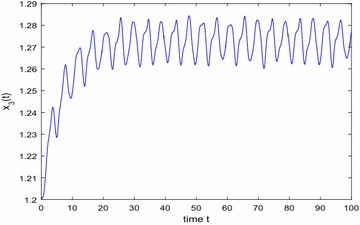
$\mathbb {T}=\mathbb {R}.$ Numerical solution $x_3(t)$ of system (7) for $(\varphi _1(0),\varphi _2(0),\varphi _3(0))=(1.2,1.25,1.2)$

**Fig. 4:**
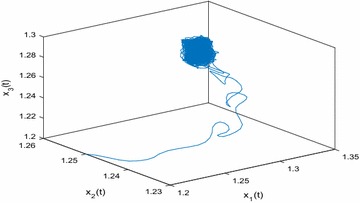
Continuous situation $$(\mathbb {T}=\mathbb {R}): x_1(t), x_2(t),x_3(t)$$

**Fig. 5:**
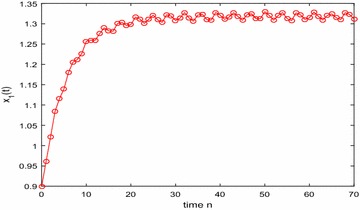
$\mathbb {T}=\mathbb {Z}.$ Numerical solution $x_1(n)$ of system (7) for $(\varphi _1(0),\varphi _2(0),\varphi _3(0))=(0.9,1.25,0.92)$

**Fig. 6:**
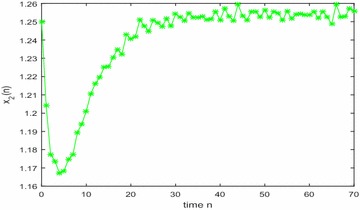
$\mathbb {T}=\mathbb {Z}.$ Numerical solution $x_2(n)$ of system (7) for $(\varphi _1(0),\varphi _2(0),\varphi _3(0))=(0.9,1.25,0.92)$

**Fig. 7:**
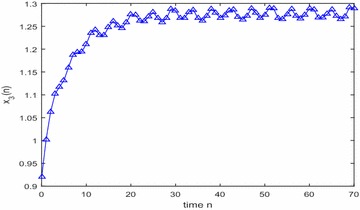
$\mathbb {T}=\mathbb {Z}.$ Numerical solution $x_3(n)$ of system (7) for $(\varphi _1(0),\varphi _2(0),\varphi _3(0))=(0.9,1.25,0.92)$

**Fig. 8:**
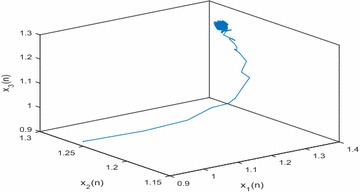
Discrete situation $$(\mathbb {T}=\mathbb {R}): x_1(n), x_2(n),x_3(n)$$

### *Remark 10*

Non of the results obtained in Chérif (2015), Duan and Huang (2015), Yao (2015a), Wang et al. (2011), Alzabut (2010), Chen and Liu (2011), Long (2012), Wang (2013), Liu and Meng (2012), Xu (2014), Ding and Alzabut (2015), Yao (2014), Alzabut (2013) can be used to obtain the results of Example 3.

## Conclusion

In this paper, we proposed a new concept of almost periodic time scales, two new definitions of almost periodic functions on time scales and investigated some basic properties of them, which can unify the continuous and the discrete cases effectively. As an application, we obtain some sufficient conditions for the existence and exponential stability of positive almost periodic solutions for a Nicholson’s blowflies model on time scales. Our methods and results of this paper may be used to study almost periodicity of general dynamic equations on time scales. Besides, based on our this new concept of almost periodic time scales, one can further study the problems of pseudo almost periodic functions, pseudo almost automorphic functions and pseudo almost periodic set-valued functions on times as well as the problems of pseudo almost periodic, pseudo almost automorphic and pseudo almost periodic set-valued dynamic systems on times and so on.
